# Design and performance of two stages square cut rectangular multiband microstrip fractal antenna

**DOI:** 10.1016/j.mex.2025.103559

**Published:** 2025-08-08

**Authors:** Sudhir Kadam, Kamalakar Ravindra Desai, Payal kadam, Aarti Prasad Pawar, Sonali Pawar, Prof. Anuradha Nigade

**Affiliations:** a Bharati Vidyapeeth (Deemed to be University) College of Engineering, Pune, India; b Bharati Vidyapeeth’s College of Engineering, Kolhapur, India

**Keywords:** Square Cut, Rectangular, Multiband, Fractal

## Abstract

Modern wireless systems require compact, low-profile, and multiband antennas. The designs offer high performance without structural complications. The classic, traditional single-band antenna mostly does not fit the bandwidth of applications. This study represented the challenges by introducing a two-stage square-cut fractal microstrip antenna design. This antenna integrated the two fractal geometries with one feed line and used periodical edge etching along with internal slotting to increase the electrical length and improve radiation efficiency without enlarging the size of the antenna. Through FEM-based simulation, the antenna dual-band characteristics for S-band (2.4263–3.2018 GHz) and C-band (5.3789–7.2308 GHz), giving a total bandwidth of approximately 3.7 GHz. The gain value remains stable about 6 dB in the S-band and 7.5 dB in the C-band, while return loss stays below –10 dB and VSWR under 2. These results offer a scalable platform for further research in reconfigurable and flexible antenna systems.

• The antenna is beneficial for 2.4263–3.2018 GHz bandwidth of approximately 1.53 GHz and 5.3789–7.2308 GHz bandwidth of approximately 1.53 GHz respectively with minimal power reflection within bands.
• Smith chart maintained at less than 51Ω. A few deviations occurred due to manual errors duringfabrication, soldering and testing antenna.

## Graphical abstract

**Figure:**
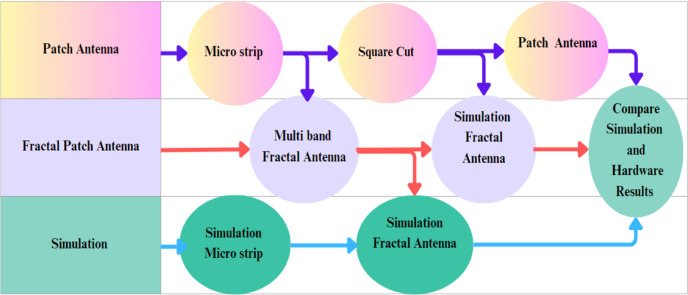

## Introduction

The swift growth in wireless communication has created immense possibilities for antennas that are to remain somewhat small and lightweight, carry out multiband functioning with high efficiency. The antennas, conceive in the wireless systems, are to transmit or receive electromagnetic signals among wireless equipment for communications, such as mobile devices, satellite systems, and IoT networks [1,2]. Microstrip patch antennas are among types of antennas widely accepted due to planar nature, low profile, and ease of integration with any popular electronic device [3]. But, conventional microstrip antennas have peculiar shortcomings: lower impedance bandwidth, low radiation efficiency, and hence poorly complement a compact design [4,5].

Fractal antenna designs have become a viable substitute to overcome these limitations. Because of their space-filling, self-similar, and recursive characteristics, fractal geometries allow for the design of antennas with longer electrical lengths in smaller physical dimensions, which improves bandwidth and multiband performance [6,7]. While traditional designs like the Koch curve and Sierpinski triangle have shown some success in this area, they frequently use single-stage configurations and might not offer the performance needed for high-frequency or complex applications [8,9]. Furthermore, as frequency requirements increase, such geometries may become structurally inefficient or challenging to optimize. These drawbacks emphasize the requirement for fractal antenna designs that are more adaptable and scalable.

By fusing sophisticated geometric structuring with performance-focused improvements, this study presents a novel two-stage square-cut fractal microstrip patch antenna that overcomes the aforementioned difficulties. The antenna's dual square-cut design, which incorporates internal cylindrical slotting and periodic edge etching, greatly lengthens the electrical path without increasing its physical dimensions. Together, these design components enhance the antenna's dual-band operation, radiation efficiency, and impedance matching. Two separate operating bands—the 2.4263–3.2018 GHz (S-band) and the 5.3789–7.2308 GHz (C-band)—are confirmed by simulations carried out using an electromagnetic solver based on the finite element method (FEM), yielding a total bandwidth of roughly 3.7 GHz. With return loss values below -10 dB and VSWR below 2, the antenna achieves a steady gain of roughly 6 dB in the S-band and 7.5 dB in the C-band.

This design's hybrid fractal geometry and capacity to achieve high performance in both the S- and C-band without sacrificing size or adding to fabrication complexity are what make it novel and significant. The suggested structure provides better multiband behavior, higher gain, and increased space efficiency in comparison to traditional patch antennas and previous fractal configurations. Further improvements like tunability, reconfigurability, or deployment on flexible substrates for wearable and embedded systems are also possible thanks to the antenna's straightforward yet scalable geometry. This work provides a solid and useful solution for next-generation communication technologies, such as 5G, IoT, defense, and satellite applications, thanks to its proven performance and efficient design [12,13].

**Specifications table**

**Table:** 

**Subject area**	
**More specific subject area**	Antenna Design
**Name of your method**	Methodology to design Two Stages Square Cut Rectangular Multiband Microstrip Fractal Antenna
**Name and reference of original method**	Not applicable
**Resource availability**	Antenna is simulated & designed using HFSS and validation using ANSYS HFSS software

## Research gap and problem statement

The design, development, and performance assessment of microstrip fractal multiband patch antennas for S-band and C-band wireless communication applications are the main topics of this research study. The study shows how dual-band patch designs and fractal geometries can be used to overcome the drawbacks of traditional antenna designs, including reduced bandwidth and efficiency loss. This discovery is positioned as a major development in antenna technology due to the incorporation of innovative features such space-filling properties, compact dimensions, and enhanced feeding processes. Applications in the 5G NR sub-6 GHz and WiMAX (2.6 GHz) bands have validated the suggested architecture, which provides a creative, scalable, and effective solution for next-generation wireless communication systems.

## Background

The need for small, high-performing antennas has increased as a result of the ongoing development of wireless technologies like 5G, the Internet of Things (IoT), satellite communications, and mobile broadband. Microstrip patch antennas are generally regarded as the best option for these kinds of applications because of their planar structure, low weight, and simplicity in integrating with printed circuit boards (PCBs). However, their use in multiband systems is limited by their conventional configurations, which frequently suffer from inherent limitations such as narrow impedance bandwidth and single-band operation. Therefore, a lot of research has been focused on changing the geometries of microstrip antennas to support multiband functionality while keeping them small and easy to fabricate [1].

The use of fractal geometries, which provide both space-filling and self-similar properties, is a useful strategy for improving antenna performance. These characteristics allow fractal antennas to increase the electrical length within a limited physical area, resulting in multiband behavior and size reduction. The advantages of recursive fractal patches for dual- and triple-band operations were illustrated by Nhlengethwa and Kumar [2]. However, a lot of fractal designs rely on intricate iterations or features at the nanoscale, which can make fabrication difficult. For example, a nano-fractal patch with dual-band performance was proposed by Kadam et al., but large-scale manufacturing is complicated by the nano-slotting technique [3]. As demonstrated by A. V. G.'s work, other studies have enhanced bandwidth and gain by utilizing Complementary Split Ring Resonators (CSRR) and Defected Ground Structures (DGS). A.V.G. et al. for WiMAX antennas [4]. However, the generalizability of these designs is limited because they frequently call for highly precise ground-plane modifications and may introduce spurious radiation.

Researchers have suggested a number of ground-plane and feed mechanism modifications to address fabrication flexibility and enhance radiation characteristics. For instance, it has been demonstrated that a slotted partial ground plane greatly improves performance in sub-6 GHz antennas for 5G applications [5]. Similar to this, the design of antipodal Vivaldi antennas with corrugated patches provides dual-polarized and wideband performance, but it has structural complexity and scalability problems [6]. In practical application, metamaterial-based and electromagnetic band gap (EBG) designs tend to compromise mechanical robustness and cost-efficiency, but they further enhance impedance matching and gain [7,8]. Furthermore, although some researchers, such as Kapoor and Kumar, concentrated on 5G wideband printed antennas, their designs frequently prioritize continuous bandwidth over selective multiband operation, which is a crucial feature for systems that need to switch between particular frequency bands [9].

A glaring research gap still exists in reaching a practical balance between performance, compactness, and fabrication simplicity in spite of these varied innovations. In order to close this gap, a novel two-stage square-cut fractal microstrip antenna is proposed in this work. In contrast to earlier methods that mainly rely on metamaterials, recursive fractals, or nano-scale slotting, the suggested design combines internal slotting, periodic edge etching, and dual square cuts to lengthen the electrical path while maintaining physical compactness. Dual-band operation in the S- and C-bands is made possible by this arrangement, which also improves gain and bandwidth without adding a lot of fabrication complexity. The suggested antenna attains a higher degree of design quality than current designs, such as the CPW-fed broadband antenna [10], mushroom-like EBG-based structures for 5G [11], and modified rectangular patches for microwave applications [12]. This positions it as a viable and scalable solution for multi-standard wireless communication systems, including mobile, IoT, satellite, and radar platforms.

By integrating key advantages of fractal geometries and structural optimization, the proposed approach fills a research gap between classical fractal methods and overly complex high-performance designs. It provides a scalable and mechanically robust antenna suitable for applications such as 5G, IoT, satellite, and defense communication systems.

To further emphasize the practical relevance, the proposed antenna directly addresses the design challenges encountered in compact multiband systems, such as size constraints, energy efficiency, and fabrication simplicity. It supports integration into portable wireless modules without sacrificing performance, making it suitable for real-world applications in mobile networks, smart devices, and satellite subsystems.

The primary research gap identified in Table 3 is that the lack of simple, dual-stage fractal microstrip designs that achieve distinct dual-band operation while maintaining low-profile geometry and ease of fabrication. Existing designs either use conventional single-stage fractals or rely on complex feed structures and ground modifications. By contrast, our proposed square-cut dual-stage structure provides an original geometry that is scalable, easy to fabricate, and delivers stable performance across targeted frequency bands.

The Patch property is added to get the results for a multiband frequency antenna. When two distinct bands with VSWRs less than two are joined, two broad bands of 2.1714 GHz and 1.7539GHz are produced, demonstrating that our antenna is effective for the specified application frequencies. Antenna performance is higher for the specified application when the frequency bands 2.2571 GHz to 3.9714 GHz and 5.000 GHz to 7.1714 GHz are distinct and the reflection coefficient is less than -10 dB.

Theory of fractal antenna

**Fig 1:**
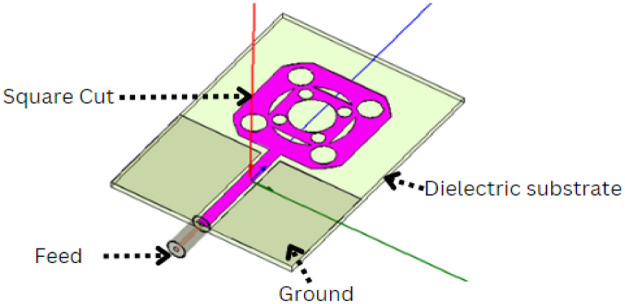
Dielectric substrate of fractal Antenna.

As seen in Fig. 3, a microstrip square-cut rectangular patch antenna is made up of a ground plane on one side and the radiating patch the other side of a dielectric substrate. The patch can take on any shape and is often composed of a conducting substance like copper or gold. Typically, the dielectric microstrip feed; feed and-stage a square-cut cut rectangular patch as shown in Fig. 1.

## Method details

**Fig 2:**
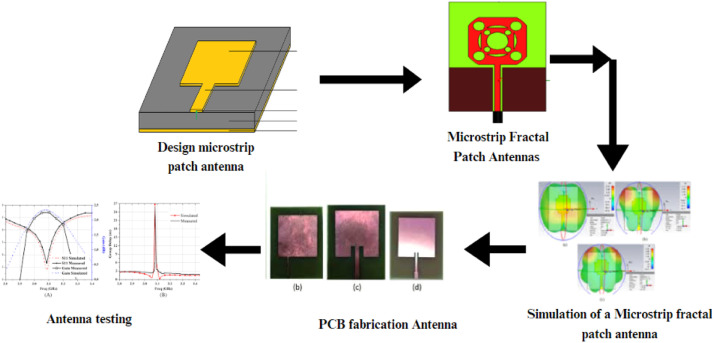
Methodology to Desin fractal Antenna.

The design of the proposed two-stage square-cut fractal antenna was carried out to support multiband operation while maintaining a compact footprint. The goal was to improve bandwidth and gain using a modified geometry that combines square-cut fractal shapes and periodic edge etching as shown in Fig. 2.

The antenna was modelled and simulated using ANSYS HFSS, a high-frequency structure simulator based on the Finite Element Method (FEM). This tool allows accurate modelling of electromagnetic fields in complex geometries and was used to evaluate return loss, VSWR, radiation patterns, and impedance matching.

The fractal antenna's multiband characteristic is demonstrated in following steps.

Step 01: Antenna a metallic apparatus commonly referred as an aerial or radiator for radio waves.

Step 02: Microstrip Antennas are commonly available inexpensive, lightweight, and conformable. These are active devices and printed strip-line feed networks. Due to their tiny size, low profile, ease of manufacture, ease of attachment to both planar and non-planar surfaces, simplicity, affordability, and mechanical robustness which is made of grounded substrate with a metallic patch, which can be square, circular, triangular, or rectangular, having adaptability for impedance bandwidth, radiation pattern, polarization, and resonant frequency. A radiating patch on one side of a dielectric substrate with a ground plane makes up a microstrip patch antenna [9].

Step 03: Fractal Antennas are low-profile portable systems available in small size for creating low-profile communication systems.

Step 04: Properties of Fractal Antennas over conventional antenna types, emitting electromagnetic energy are found important for Fractal shapes. By utilizing the fractal geometry for traditional antenna configuration decreasing antennas' size and optimizing shape to enhance their electrical length. With two primary characteristics of fractal geometries—space-filling and self-similar properties—fractal shape antenna elements offer a number of benefits, including multiband, broad bandwidth, and smaller antenna sizes [11].

Step 05: Geometry Fractal antennas are more effective than patch antenna size reduction. By optimizing the geometry, filling the patch's edges with inductive components, etching periodic slow wave patterns on the ground plane, and inserting slots into the patch, the shorting posts procedures increase the electrical length of the antenna.

Step 06: Optimization of Fractals antennas are self-similar geometrical structures, by getting repeated for specific simple geometries, can produce nearly any complex structure found in nature. Extending the antenna's overall electrical length, it increases the area of material utilized in the antenna.

Step 07: Scaled Down Fractal antennas recurrence of a motif over two or more scale sizes, or "iterations.", known as multilevel and space filled curves. These are incredibly small, multiband or wideband, and have practical uses in microwave and cellular phone communications.

Step 08: Simulation Results Fractal antennas the antenna exhibits discrete bands between given frequencies. Additionally, its reflection coefficient, gain VSWR to indicating superior performance for the specified application.

## Method validation

When creating an antenna, the Patch property is added to get the results for a multiband frequency antenna. When two distinct bands with VSWRs less than two are joined, two broad bands of 2.1714 GHz and 1.7539GHz are produced, demonstrating that our antenna is effective for the specified application frequencies in Table 2– Frequency range for both bands. Antenna performance is higher for the specified application when the frequency bands 2.2571 GHz to 3.9714 GHz and 5.000 GHz to 7.1714 GHz are distinct and the reflection coefficient is less than -10 dB. The 8-pattern, indicating that the antenna is bidirectional.

Small Size: It is quite small. This antenna's primary benefit is its ability to be adhered to the surface where it will be utilized, which saves space and makes it appear appealing because of its self-similarity. The size in relation to antennas will determine the gain and efficiency [15].

Space Filling Property: Because the system can operate in several bands with the same size of antenna, it makes efficient use of the available area. It boasts outstanding improvements in efficiency and is quite small [14].

**Fig 3:**
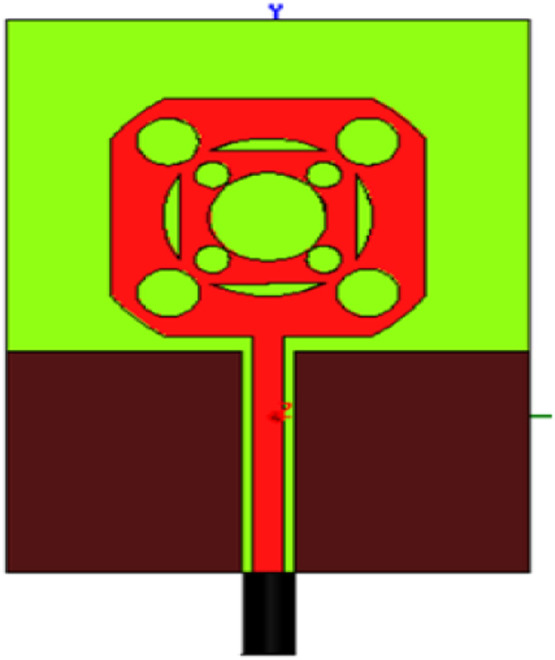
Multiband Property explanation of fractal Antenna.

Mechanical Simplicity and Robustness: Discrete components' shape determines the antenna's properties. It is excellent for applications such as space satellite applications since it can be adhered to any surface [12]. Above is the demonstration of the self-similar property of the antenna. The same basic rectangular shape is repeated over each time in Fig. 3.

## Mathematical modelling

Step 1: Calculation of the Width (W):

The width of the Microstrip patch antenna is given as:

$$W=\frac{C}{2f_{r}\sqrt{\left(\frac{\varepsilon_{r}+1}{2}\right)}}$$

Where; c - Free space velocity of light, 3 × 108 m/s fr - Frequency of operation

εr - dielectric constant

Step 2: Calculation of Effective dielectric constant (εreff):

The effective dielectric constant is:

$$\varepsilon_{\text{reff}}=\frac{\varepsilon_{r}+1}{2}+\frac{\varepsilon_{r}-1}{2}{\left\{1+12\frac{h}{w}\right\}}^{1/2}$$

Where;

εr - Dielectric constant h - Height of dielectric substrate

W - Width of the patch

Step 3: Calculation of the Effective length (Leff):

The effective length is:

$$L_{\text{eff}}=\frac{C}{\;2f_{r}\sqrt{\varepsilon_{\text{reff}}}}$$

Where; c - Free space velocity of light, 3 × 108 m/s fr - frequency of operation

εreff - effective dielectric constant

Step 4: Calculation of actual length of patch (L):

The actual length is obtained by:

$$\;L=L_{\text{eff}}-2\Delta L$$

Where,

L= Actual length of patch.

Leff= Effective length.

∆L= small difference between length.

Step 5: Calculation of the length extension (∆L):

The length extension is:

$$\Delta L=0.412h\frac{\left(\varepsilon_{\text{reff}}+0.3\right)\left(\frac{W}{h}+0.264\right)}{\left(\varepsilon_{\text{reff}}-0.258\right)\left(\frac{W}{h}+0.8\right)}$$

The radiating antenna is created using a rectangular patch of 28 mm x 28 mm dimensions as shown in Fig. 3 on a rectangular FR4 substrate of size 48 mm x 68 mm. Height = 1.53 mm and permittivity = 4.4. This basic structure (square cut) is then curved at the edges by intersecting with a cylinder of radius 18 mm, whose center is the centroid of the square. Curved edges help in the improvement of bandwidth operation. The central part of the cylindrical shape of 10 mm is etched out.

The fractal property is added to get the results of two distinct frequency bands of 2.1714 GHz and 1.7143 GHz that are produced. The reflection coefficient is less than -10 dB, indicating that the antenna is bidirectional, achieving high gain for the operational frequency band. Having a width of 3.1 mm. coplanar ground on both sides of the feed with size 21.5 mm x 27 mm. The gap between ground & feed is 0.95 mm with ground to patch 2 mm as described in Table 1.

**Table 1:** Speciation of designed antenna.

Name	Adjusted Value	Equations Value
rectangular patch	28 mm x 28 mm	30 mm x 30 mm
substrate	48 mm x 68 mm.	49 mm x 70 mm.
cylinder of radius	18 mm	18 mm
width	3.12 mm	3.15 mm
Height	1.53 mm	1.65 mm
gap between ground & feed	0.95 mm	0.98 mm

**Table 2:** Frequency range for both bands.

Starting frequency in GHz	Ending frequency in GHz	frequency band in GHz
2.2175	3.9714	1.7539
5.0000	7.1714	2.1714

**Table 3:** Comparison between different characters with feeding techniques.

Characteristics	Microstrip Line Feed	Coaxial feed	Aperture coupled feed	Novelty of the research
Spurious feed radiation	More	More	Less	Minimum
Reliability	Better	Poor due to soldering	Good	Good
Ease of fabrication	Easy	Soldering and drilling needed	Alignment required	Alignment required
Impedance matching	Easy	Easy	Easy	Easy
Bandwidth	2-5% Ref[1],[3]	2-5% Ref[8],[12]	2-5% Ref[10],[14]	13%

This antenna design and geometry allow it to function at a variety of frequency bands. Fractal antennas save both hardware and electricity. Small in size, benefitting the determination related to antennas gain and efficiency. Operating in several bands with the same size of antenna, it makes efficient use of the available area.

Highlights

VSWR should be less than 2, means that the antenna is operating in S band and C band. Results are obtained for distinct bands in each Antenna type.

The Smith chart is maintained at less than 51Ω. A few deviations occurred due to manual errors during fabrication, soldering and or testing of antenna.

## Simulated results

A technique for large structures is converted into multiple fractal-shaped structures for ease of analysis. Reflection coefficient measures electromagnetic wave reflection in the transmission channel bringing impedance discontinuity. Frequencies below -10dB yielding the return loss bandwidth. There is plotted gain of the directive angles varying from 0^o^ to 360^o^ The angle deviates from the original plane, decreasing gain. Antenna gain was found uniformly around 6 dB for the entire operating band of 2-4 GHz and uniform around 7.5 dB for the entire operating band of 6-8 GHz. The overall nature of the graph is increasing from lower to higher bands for all antennas.

The software is based on the FEM method; in this technique, large structures are converted into multiple fractal-shaped structures for ease of analysis.

## VSWR

**Fig 4:**
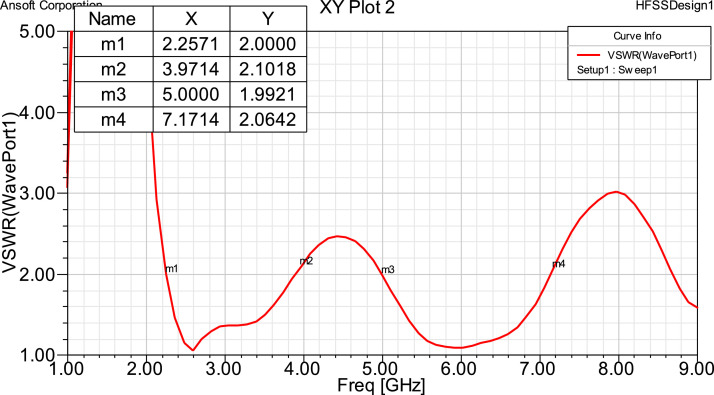
VSWR for Fractal antenna.

The proposed antenna to be in the operating band, VSWR should be less than 2. As seen from the following simulation results, we get 2 distinct bands in each antenna. By showcasing the manufactured antenna's impedance-matching properties and resonant behavior over the operational frequency ranges, the experimental VSWR measurements, as displayed in Fig. 4(b), showcase its performance in real-world scenarios. At the resonant frequency of 2.2 GHz, the graph shows a VSWR value below 2, indicating efficient operation with low power reflection and ideal impedance matching. This outcome validates the antenna design and production process because it closely matches the simulated data.

## Reflection coefficient

**Fig 5:**
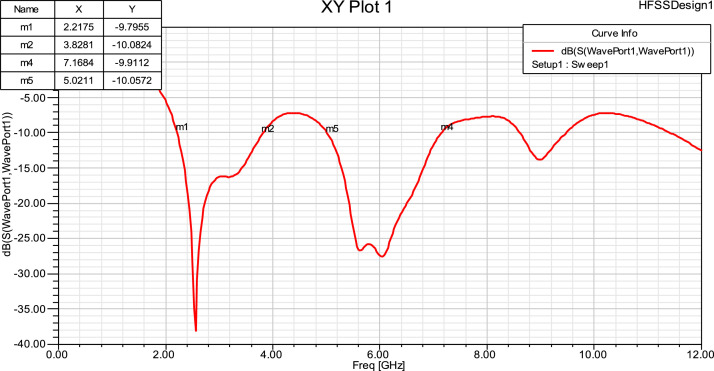
Reflection Coefficient for Fractal antenna.

The amount of reflection coefficient measures electromagnetic wave reflection in the transmission channel brought on by an impedance discontinuity. This is the ratio of the amplitude of the incident wave at the intersection to that of the reflected wave. Deducing frequencies below -10dB yields the return loss bandwidth. Fig. 5 shows that the return loss graph agrees with the VSWR findings.

The graph shows dips below -10 dB at multiple frequencies, confirming the antenna's operational bands with effective impedance matching. Specifically, return loss values of -10.0586 dB at 2.2 GHz, -10.0741 dB at 3.8 GHz, -9.7359 dB at 5.02 GHz, and -10.2599 dB at 7.16 GHz are observed. These results correspond to the antenna's designed S-band and C-band frequency ranges, ensuring minimal power reflection within these bands.

## Radiation pattern

**Fig 6:**
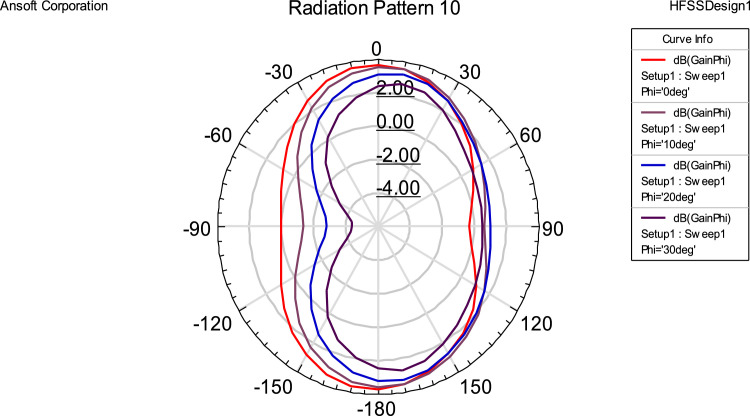
Radiation Pattern for Fractal antenna.

Antenna has a bi-directional pattern as shown in Fig. 6. The pattern can be plotted as a function of radiated energy, directivity, electric field intensity, magnetic field distribution, gain, etc. For our project, we haveplotted gain as a function of directive angles varying from 0^0^ to 360^0^. In antennas, as the angle deviates from the original plane, gain decreases. This yields a bidirectional pattern like the figure of Eight (8).

The readings taken at different angles of elevation (e.g., θ = 0°, 10°, 20°, and 30°). The findings show that the gain diminishes with increasing angle deviation from the reference plane, suggesting that radiation intensity reduces with increasing angular displacements. The pattern's symmetrical and almost uniform distribution highlights the antenna's performance stability and consistency, which are critical for dependable wireless communication. The "figure-eight" design indicates that it is bi-directional, which is beneficial for applications like point-to-point communication systems that need targeted coverage along a particular plane. These findings support the antenna's design by demonstrating its capacity to provide regulated radiation characteristics and strong directivity.

## Peak gain

**Fig 7:**
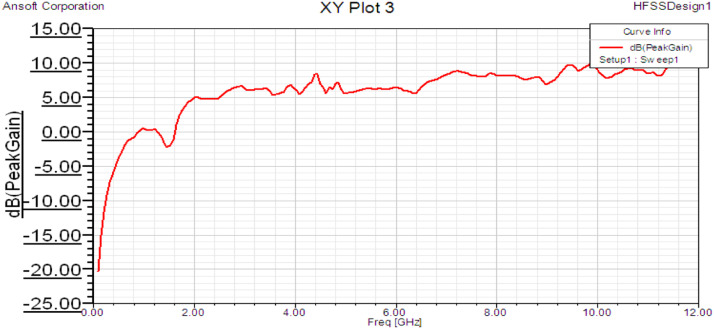
Peak gain for Fractal antenna.

The antenna gain was found to be uniform around 6 dB for the entire operating band of 2-4 GHz and uniform around 7.5 dB for the entire operating band of 6-8 GHz. It is observed that the overall nature of the graph is increasing from lower to higher bands for all antennas. The further graph is exponentially increasing for 0-2 GHz and is linearly increasing after 2 GHz, as shown in Fig. 7. The gain of less than 0 indicates non-radiating frequencies.

## Smith chart

This chart is a vital tool for evaluating the antenna's impedance matching and reflection behavior. At 1.955 GHz, the impedance is approximately 51.80 Ω – j19.65 Ω, corresponding to a capacitive reactance of 4.14 pF, which is close to the ideal value of 50 Ω. This indicates good matching and efficient energy transfer at this frequency.

**Fig 8:**
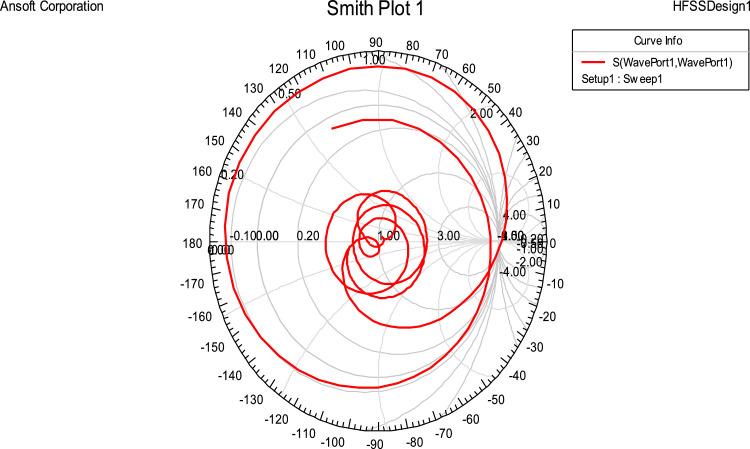
Smith chart of fractal antenna.

Following Fig. 8 shown that at 1.95GHz, impedance is 51Ω. Both the results are in almost the same agreement; minor deviations occurred due to manual errors during fabrication, soldering, and/or testing of the antenna.

## 3D radiation patterns

**Fig 9:**
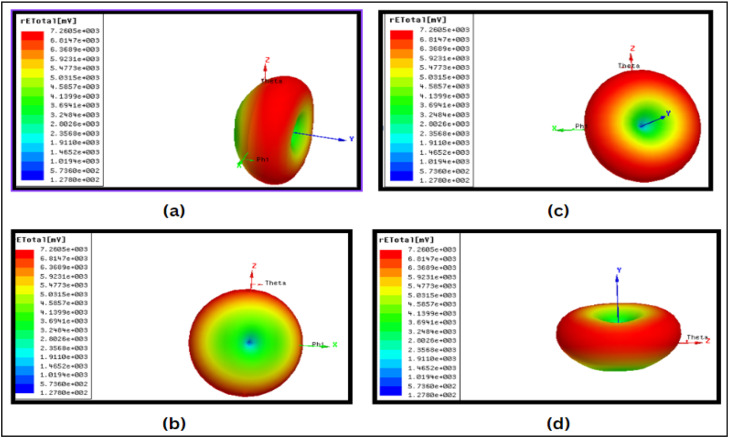
3D radiation patterns of the proposed antenna.

The 3D radiation patterns of the proposed MPA at various directions and frequency bands, specifically in the S-band of 2.2 GHz to 3.8 GHz and C-band of 5.02 GHz to 7.22 GHz. The patterns depict the spatial distribution of the radiated electromagnetic energy with color-coded intensity levels, where red indicates maximum radiation intensity and blue represents minimum radiation intensity as shown in Fig. 9.

## Surface current distribution

**Fig 10:**
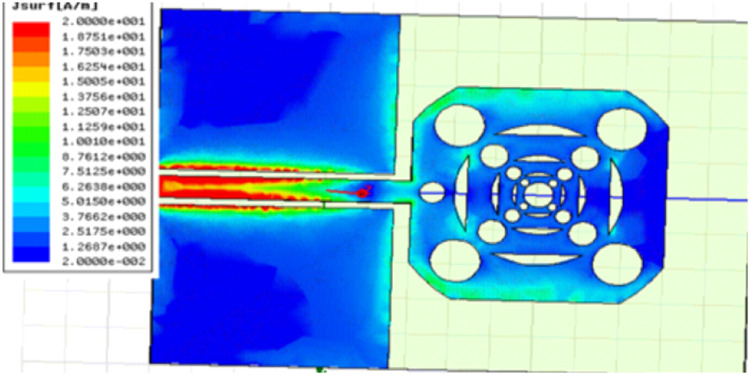
Current Distribution of the proposed antenna.

With a careful design that produces effective results in VSWR, Smith Chart, and Reflection Coefficient evaluations, the simulation is run for frequency ranges up to 7 GHz. Modern wireless communication systems depend on steady multiband operation, reduced energy losses, and improved radiation efficiency, all of which are supported by the optimal current distribution as shown in Fig. 10.

## Conclusion

The simulation results demonstrate two distinct frequency bands of operation: the first band extends from 2.4263 GHz to 3.2018 GHz (S-band), providing a bandwidth of approximately 1.53 GHz, while the second band spans 5.3789 GHz to 7.2308 GHz (C-band), offering a bandwidth of approximately 2.17 GHz. This dual-band performance results in a total operating bandwidth of approximately 3.7 GHz, making the antenna highly suitable for S-band and C-band wireless communication applications. A unique two-stage square-cut microstrip patch antenna has been designed and studied in this work in response to the growing need for truly compact multiband solutions in wireless communication systems. The dual-band operation was successfully triggered by geometrical modifications that increase the electrical length of the antenna as well as improve its radiation characteristics. Simulation results reveal that the antenna can work well over S- and C-band frequencies with return loss less than -10 dB and VSWR less than 2. These parameters are sufficient for an antenna to be practical in the wireless arena.

The square-cut geometry has been selected to obtain a multiband antenna while maintaining the mechanical simplicity and compactness of the antenna footprint. Further periodic edge etching and slotting increase bandwidth and gain performance without adding bulk to the antenna.

Looking ahead, this design lays the groundwork for further research in fractal-based antenna miniaturization. Future work may involve fabricating the antenna and experimentally validating the simulated results, integrating reconfigurable or tunable components to allow dynamic frequency agility, or applying the design concept to flexible substrates for wearable or embedded IoT systems. With ongoing enhancements, this approach can contribute significantly to the development of next-generation antennas for applications ranging from 5G to satellite and defense communication networks.

## Supplementary material and/or additionalinformation [OPTIONAL]

“None”.

## Declaration of competing interest

The authors declare that they have no known competing commercial interests or personal relationships that could have appeared to influence the work reported in this paper.

## Data Availability

Data will be made available on request.

## References

[1] Nhlengethwa N.L., Kumar P. (2021). Fractal microstrip patches antennas for dual-band and triple-band wireless applications. Int. J. Smart Sens. Intell. Syst..

[2] Kadam S., Gurav S., Patil V., Jadhav P., Prabhakar A.Y., Thorat H.S. (2024). Design and Performance of Dual Band Microstrip Nano-Fractal Patch Antenna. Nanotechn. Percept..

[3] A V.G., P A.R., Mathew T. (2019). Microstrip antenna with DGS based on CSRR array for WiMAX applications. Int. J. Electr. Comput. Eng..

[4] Kapoor A., Mishra R., Kapoor A., Kumar P. (2020). Compact wideband-printed antenna for sub-6 GHz fifth-generation applications. Int. J. Smart Sens. Intell. Syst..

[5] Kumar Sumit, Dixit Amruta, Choubey Chandan (2024). Design of Antipodal Vivaldi Antenna with Patch and Corrugations for 5G Applications. MethodsX..

[6] K. Desai, “Design of Dual Band Microstrip Fractal Antenna Design of Dual Band Microstrip Fractal Antenna,” no. June 2023.

[7] TY - JOURT1. Achieving desired characteristic impedances in customized coplanar waveguide transmission line designAU - Alrashdan, Mohd H. SJO - MethodsXVL - 13SP - 103074PY - 2024DA - 2024/12/01/SN - 2215-0161DO 10.1016/j.mex.2024.103074. PMC11663961 39717124

[8] sharma Vijay, sharma Brajraj, sharma K.B., saxena V.K. (2005). D. bhatnagar “a novel dual frequency s -band rectangular microstrip antenna for radar and space communication. J. Theoretic. Appl. Inform. Technol..

[9] Lin Shu, Qiu Jinghui, Ren He (Nov 2007).

[10] Varshney Leena, Gupta Vibha Rani, Kumar Harish, Suraj Priyadarshi (Jan 2011). CPW-Fed Broadband Microstrip Patch Antenna. Publish. Int. J. Adv. Eng. Applic..

[11] Alsudani Ahlam, Marhoon M. (2023). Design and Enhancement of Microstrip Patch Antenna Utilizing Mushroom Like-EBG for 5G Communications. J. Commun..

[12] Marhoon M., Abdulnabi Hussein, Al-Aboosi Yasin. (2022). Designing and Analysing of a Modified Rectangular Microstrip Patch Antenna for Microwave Applications. J. Commun.J. Commun..

